# An investigation of breast cancer risk factors in Cyprus: a case control study

**DOI:** 10.1186/1471-2407-10-447

**Published:** 2010-08-23

**Authors:** Andreas Hadjisavvas, Maria A Loizidou, Nicos Middleton, Thalia Michael, Rena Papachristoforou, Eleni Kakouri, Maria Daniel, Panayiotis Papadopoulos, Simon Malas, Yiola Marcou, Kyriacos Kyriacou

**Affiliations:** 1 Department of EM/Molecular Pathology, The Cyprus Institute of Neurology and Genetics, Nicosia, Cyprus; 2 Department of Nursing, School of Health Sciences, Cyprus University of Technology, Nicosia, Cyprus; 3 Bank of Cyprus Oncology Center, Nicosia, Cyprus; 4 Department of Oncology, Limassol General Hospital, Limassol, Cyprus

## Abstract

**Background:**

Breast cancer is the most common form of malignancy affecting women worldwide. It is also the leading cancer in females in Cyprus, with approximately 400 new cases diagnosed annually. It is well recognized that genetic variation as well as environmental factors modulate breast cancer risk. The main aim of this study was to assess the strength of associations between recognized risk factors and breast cancer among Cypriot women. This is the first epidemiological investigation on risk factors of breast cancer among the Cypriot female population.

**Methods:**

We carried out a case-control study, involving 1,109 breast cancer patients and a group of 1,177 controls who were recruited while participating in the National screening programme for breast cancer. Information on demographic characteristics and potential risk factors were collected from both groups during a standardized interview. Logistic regression analysis was used to assess the strength of the association between each risk factor and breast cancer risk, before and after adjusting for the possible confounding effect of other factors.

**Results:**

In multivariable models, family history of breast cancer (OR 1.64, 95% CI 1.23, 2.19) was the strongest predictor of breast cancer risk in the Cypriot population. Late menarche (OR 0.64, 95% CI 0.45, 0.92 among women reaching menarche after the age of 15 vs. before the age of 12) and breastfeeding (OR 0.74, 95% CI 0.59, 0.92) exhibited a strong protective effect. In the case of breastfeeding, the observed effect appeared stronger than the effect of pregnancy alone. Surprisingly, we also observed an inverse association between hormone replacement therapy (HRT) although this may be a product of the retrospective nature of this study.

**Conclusion:**

Overall the findings of our study corroborate with the results of previous investigations on descriptive epidemiology of risk factors for breast cancer. This investigation provides important background information for designing detailed studies that aim to improve our understanding of the epidemiology of breast cancer in the Cypriot population, including the study of gene-environment interactions. Furthermore, our study provides the first scientific evidence for formulating targeted campaigns for prevention and early diagnosis of breast cancer in Cyprus.

## Background

Breast cancer is the most frequent malignancy in the female population of Europe and North America where an estimated 1 in 9 women are at risk of developing the disease [1]. Numerous epidemiological studies over the last three decades have revealed a number of risk factors associated with breast cancer [2-4]. In addition to genetic and reproductive factors, breast cancer risk displays wide ethnic and geographic variation [5]. Within Europe the incidence varies by almost two-fold. It is highest in Northern European countries, with an estimated 84.6 cases per 100,000 population with intermediate rates recorded in Southern Europe and lowest rates occurring in Eastern Europe with 42.6 cases per 100,000 population [6]. In addition to the geographic variation, influences on incidence rates, have also been attributed to differences in the use of mammography, diet, physical activity, body size, alcohol consumption and socioeconomic and reproductive factors [7]. The discovery of highly penetrant breast cancer susceptibility genes such as the *BRCA *genes in the mid 1990's [8,9] emphasized the importance of genetic factors, but it is currently believed that environmental factors are of greater significance [10].

Cyprus is the third largest island in the Mediterranean Sea with a Greek-Cypriot population of 749,200 as recorded in July 2004, by the statistical service of the Republic of Cyprus. Data from the National Cancer Registry established in 1998 by the Ministry of Health show an average incidence of 400 female breast cancer cases per year [11]. This corresponds to an age-standardized incidence rate of breast cancer of 60 per 100,000 population [11] which is comparable to the rates seen in other Southern European countries, a region where a moderate breast cancer risk is usually recorded [6]. Cyprus is a member of the European Union since 2004, and has a long history of practicing a Mediterranean lifestyle. However the lifestyle is rapidly changing towards a western type of life, so it is of interest to study the pattern as well as the influence of breast cancer risk factors in our population.

Indeed, there are no data as yet available about breast cancer risk factors in the Cypriot female population. The main aims of this report are (a) to describe the background levels of established and recognized breast cancer risk factors among Cypriot women and (b) to assess the strength of their association with breast cancer risk. In order to achieve this we carried out a case-control study, involving a breast cancer patient group and a control group of healthy Cypriot women attending mammography clinics as part of the National screening programme. A number of risk factors that modulate breast cancer such as socioeconomic status, reproductive events, family history and lifestyle factors were investigated.

Interestingly, previous published work by our group has shown that the Cypriot population exhibits some unique genetic features as revealed by the identification of novel mutations in the *BRCA1 *and *BRCA2 *genes [12,13]. Moreover, this is the first study into breast cancer epidemiology among the Cypriot female population and will function as a baseline, of future efforts into combining person-based data, with the molecular/genetic data in order to study gene-environment interactions in our population.

## Methods

### Study participants and data collection

The sample size was determined using power calculations in order to ensure at least 90% power to detect a magnitude of association at the odds ratio scale of 1.5 at 5% statistical significance for an exposure that occurs among 10% of the controls. The cases consisted of 1109 women, between 40-70 years of age who had a histologically confirmed diagnosis of primary breast cancer between January 1999 and December of 2005. Younger cases were excluded since in addition to the fact that early-onset breast cancer may share a different aetiology, the source of the controls (i.e. mammography screening users) only effectively covers women in this age range. During the study period (January 2004 - December 2006), breast cancer cases that fulfilled the above criteria were approached while waiting for their oncologist appointment and were given an introductory letter outlining the goals of the study. Those who agreed to participate in the study subsequently underwent a personal interview with a trained interviewer. The majority of cases were ascertained from the Bank of Cyprus Oncology Centre, which operates as a referral centre and offers treatment and follow-up for up to 90% of all breast cancer cases diagnosed in Cyprus. The rest of the patients, were recruited at the Oncology Departments of the Nicosia, Limassol, Larnaca and Paphos district hospitals.

The control group consisted of 1177 Cypriot women from the general population, who were invited to participate in the National programme for breast cancer screening with the use of mammography and received a negative result. Volunteers were enrolled in the study during the same calendar period as the cases, from the 5-district mammography screening centers that operate in Cyprus. Due to the narrow age span of women invited for screening (ages 50-69) younger and older women were underrepresented. Nevertheless, between the ages of 50-65, the observed age distribution roughly matched the age structure of the island's female population. Women were approached while waiting for their mammography appointment and were given an introductory letter outlining the goals of the study. Those who agreed to participate in the study subsequently underwent a personal interview with a well trained interviewer.

Demographic and risk factor data were collected from both cases and controls with the use of a specially designed questionnaire, through a standardized interview. The questionnaire used in the present study was primarily based on a similar questionnaire used in the East Anglia breast cancer study [14]. The study was approved by the Cyprus National Bioethics Committee. Each participant gave written consent and the data were anonymised and coded into an MS ACCESS database. The questionnaire included information on age, marital status, level of education, current BMI, exercise status, smoking status (i.e. never, past, current smokers), family history of breast cancer (i.e. first degree relatives), use of hormone replacement therapy and a range of reproductive characteristics such as age at menarche, any pregnancy, gestation period, parity, overall duration of breastfeeding, use of oral contraceptives, age at first and last pregnancy (at the beginning of the term). Other than BMI (which was based on actual measurements of weight and height), all other factors investigated here, were self-reported. Only pregnancies with a gestation period of at least 24 weeks were considered (including those not resulting in a live birth). Only use of oral contraceptives or HRT for at least a period of 1 month was considered. Exercise was taken to mean any activity involving a minimum of 30 minutes walk, or other form of regular exercise including cycling, swimming, jogging, etc at least 3 times a week for the past 6 months. Type of occupation was not taken into account as a form of exercise.

### Data Analysis

We computed frequencies of categorical variables (or means and standard errors in the case of continuous variables such as age) separately for cases and controls. The frequencies were cross-tabulated and differences in participant characteristics between cases and controls were statistically assessed using the χ^2 ^test. The strength of association between each factor and breast cancer risk was estimated by odds ratios (ORs), calculated using logistic regression before and after adjusting for the confounding effect of all other risk factors, in multivariable models. In the case of ordered categorical variables (e.g. age at menarche), p-value for linear trend was reported. Due to the narrow age span of women invited for screening (ages between 50 and 69), women younger than 45 or older than 65 were under-represented in the study. To control for age differences between cases and controls, all model estimates (including associations with single risk factors) were therefore adjusted for age. Furthermore, the effect of restricting the investigation to women between 45-64 years of age was also tested in sensitivity analyses. In all cases, the fit of the models was assessed on the basis of the Pearson χ^2 ^and/or Hosmer-Lemeshow goodness-of fit statistics (which compare the observed against the expected counts as estimated by the model) producing a non-significant result.

A number of characteristics investigated here are (a) dependent on each other such as pregnancy and breastfeeding, or (b) are nested in each other such as breastfeeding status vs. breastfeeding duration or (c) are expected to display a high degree of collinearity i.e. number of children and breastfeeding. The inter-relationships between these variables and the extent to which they are independently associated with breast cancer risk was firstly assessed in separate analyses (using stratification techniques or multivariable logistic models as appropriate), with the aim of identifying appropriate course of action e.g. include the variable with the strongest effect in order to avoid multicollinearity in the final model. For instance, the possible protective effect of pregnancy and the added effect of breastfeeding over the effect of pregnancy was investigated in a logistic regression model that included the main effect of pregnancy and an interaction term between pregnancy and breastfeeding (to capture the combined effect of the two variables) but no separate main effect for breastfeeding (since only women who have had a child breastfed). Similarly, as women with more children may be more likely to breastfeed (and in fact breastfeed for longer), we assessed the extent to which breastfeeding confounds or modifies any observed association between breast cancer risk and number of children by calculating Mantel-Haenszel odds ratios after stratifying by breastfeeding status along with a χ^2 ^test of heterogeneity of estimates. All data manipulation and statistical analyses were performed in STATA SE 9.0.

## Results

A total of 2286 women - 1109 breast cancer cases and 1177 healthy controls (98% and 98% participation rate respectively) - from all five districts of Cyprus participated in the study. The mean ± SD age of cases and controls were 56.1 ± 9.1 and 55.7 ± 7.0 years respectively (p-value of t-test for difference in means = 0.22). Nevertheless, differences in the age distribution of cases and controls recruited into the study were observed mainly due to the under-representation of younger and older age-groups amongst the controls. Table 1 presents the distribution of socio-economic characteristics and potential risk factors investigated among cases and controls separately. One in four Cypriot women have never breast-fed, as many as 80% have had at least two children and more than 70% reported having never used hormone replacement therapy. No statistically significant differences between cases and controls were observed in terms of level of education, marital status, number of children or use of oral contraceptives. In contrast, a higher proportion of cases than controls reported family history of breast cancer (14.1% vs. 9.1%) and early menarche (14.2% vs. 11.4%).

**Table 1:** Socio-demographic characteristics and potential risk factors among participants.

Characteristic	Cases (N = 1109)		Controls (N = 1177)		
		
	N	%	N	%	**p-value**^**1**^
Age					
<45	121	10.9	68	5.8	
45-54	354	31.9	440	37.4	
55-64	394	35.5	526	44.7	
≥65	240	21.6	140	11.9	
Not reported	0	0.0	3	0.3	<0.01^2^
*Mean (SD)*	*56.1 (9.1)*		*55.7 (7.0)*		*0.22*^*3*^

Education					
Primary	418	37.7	464	39.4	
Secondary	412	37.2	435	37.0	
Tertiary	131	11.8	147	12.5	
University	135	12.2	122	10.4	
Not reported	13	1.2	9	0.8	0.51

Marital status					
Married	921	83.1	1015	86.2	
Widowed/Divorced	130	11.7	117	9.9	
Single	57	5.1	42	3.6	
Not reported	1	0.1	3	0.3	0.09

BMI					
<18.5	13	1.2	11	0.9	
18.5-24.5	314	28.3	322	27.4	
25-29.5	368	33.2	463	39.3	
≥30	337	30.4	269	22.9	
Not reported	77	6.9	112	9.5	<0.001

Family history of breast cancer					
No	953	85.9	1068	90.7	
Yes	156	14.1	107	9.1	
Not reported	0	0.0	2	0.2	<0.001

Age at menarche					
≤11 years	157	14.2	134	11.4	
12-14 years	798	72.0	838	71.2	
≥15 years	154	13.9	205	17.4	0.02

Pregnancy^4^					
Yes	1021	92.1	1110	94.3	
No	88	7.9	67	5.7	0.03

Number of children^4^					
None	103	9.3	88	7.5	
One	90	8.1	82	7.0	
Two	480	43.3	492	41.8	
Three or more	436	39.3	515	43.8	0.10

Age at 1st pregnancy^5^					
<30	851	83.4	971	87.5	
≥30	140	13.7	116	10.5	
Not reported	30	2.9	23	2.1	0.03

Breastfeeding^5^					
Never	305	29.9	279	25.1	
≤6 months	329	32.2	385	34.7	
7-12 months	166	16.3	181	16.3	
More than 1 year	221	21.7	265	23.9	0.09

Oral contraceptive use					
No	823	74.2	879	74.7	
Yes	280	25.3	294	25.0	
Not reported	6	0.5	4	0.3	0.76

HRT use					
Never	937	84.5	838	71.2	
<6 months	41	3.7	64	5.4	
6-60 months	86	7.8	182	15.5	
≥60 months	45	4.1	92	7.8	
Not reported	0	0.0	1	0.1	<0.001

Smoking					
Never	869	78.4	955	81.1	
Past	130	11.7	76	6.5	
Current	108	9.7	143	12.2	
Not reported	2	0.2	3	0.3	<0.001

Exercise^6^					
Yes	544	49.1	635	54.0	
No	563	50.8	539	45.8	
Not reported	2	0.2	3	0.3	0.06

**Notes**: ^1^P-value of χ^2 ^test for independence. ^2^Differences in the age distribution among cases and controls are mainly driven by the under-representation of controls in the younger and older age-groups. After restricting to ages 45-65, p-value becomes 0.46. ^3^P-value of t-test for difference in mean age between cases and controls. ^4^The difference in number of women reporting not having had a pregnancy (N = 155) and not having had a child (N = 191) is due to the fact that 15 and 21 women in cases and controls respectively have reported been pregnant not resulting in birth. ^5^Restricted to those women who have had a pregnancy. ^6^Only information on current exercise status was available.

Some strong associations were observed between the reproductive factors investigated and breast cancer risk. In general, cases were less likely than controls to have been pregnant (OR 0.69, 95%CI 0.49-0.97) or to have breastfed (OR 0.69, 95%CI 0.57-0.82). These variables are expected to exhibit a high degree of collinearity. Table 2 investigates the synergistic role of (a) breastfeeding and pregnancy as well as (b) breastfeeding and number of children in breast cancer risk. The combined effect of breastfeeding and pregnancy appeared stronger than the effect of pregnancy alone; the association with the latter attenuated after accounting for breastfeeding. Interestingly, no stepwise relationship was observed between breast cancer risk and reported duration of breastfeeding. In fact, risk of breast cancer appeared reduced across all categories of duration of breastfeeding while estimated odds ratios were of similar magnitude. The Likelihood Ratio Test comparing the goodness of fit of the model with ordered categories vs. the simpler model where breastfeeding was expressed as a binary variable produced p-values of 0.27 in the unadjusted, 0.19 and 0.33 in the adjusted models respectively, suggesting no evidence of an improved fit of the full model. Further adjusting for all other variables did not alter our inferences.

**Table 2:** Odds Ratios of breast cancer risk in terms of A) pregnancy and breastfeeding (either status or duration) and B) breastfeeding and number of children before and after adjusting for the effect of each other and all other risk factors in multivariable logistic models

	Before adjustment		After mutual adjustment		Fully adjusted^4^	
	
	OR (95%CI)	p-value	OR (95%CI)	p-value	OR (95%CI)	p-value
A. Pregnancy						
No	1.00					
Yes	0.69 (0.49,0.97)	0.03	0.89 (0.62,1.28)	0.54	1.07 (0.66,1.72)	0.79
Breastfeeding^1^						
No	1.00					
Yes	0.69 (0.57,0.82)	<0.001	0.63 (0.45,0.89)	<0.01	0.72 (0.59,0.90)	<0.01
Breastfeeding by duration^1, 2^						
Never	1.00					
≤6 months	0.71 (0.58,0.88)		0.65 (0.45,0.94)		0.73 (0.57,0.93)	
7-12 months	0.76 (0.58,0.98)		0.69 (0.47, 1.03)		0.86 (0.64,1.16)	
>1 year	0.61 (0.48,0.77)	<0.001^3^	0.56 (0.38,0.81)	<0.001^3^	0.68 (0.51,0.91)	0.04^3^

B. Breastfeeding						
No	1.00					
Yes	0.69 (0.57,0.83)	0.03	0.72 (0.58,0.88)	<0.01	0.72 (0.58,0.90)	<0.01
Number of children						
None	1.00					
One	0.90 (0.59,1.37)		1.06 (0.69,1.64)		1.29 (0.76,2.18)	
Two	0.84 (0.61,1.15)		1.04 (0.74,1.48)		1.27 (0.76,2.18)	
Three	0.68 (0.49,0.94)	<0.01^3^	0.90 (0.62,1.29)	0.25^3^	1.14 (0.82,1.99)	0.87

**Notes**: ^1^In the adjusted models, odds ratio refers to the combined effect of pregnancy and breastfeeding, status or duration respectively, as estimated in a model with an interaction term between the two variables but no main effect for breastfeeding. ^2^p-values of Likelihood Ratio Test comparing the goodness of fit of model including breastfeeding duration Vs simpler model with breastfeeding expressed as binary variable = 0.27, 0.19 and 0.33 respectively, indicating no better fit of full model. ^3^P-value for trend across ordered categories. ^4^Due to collinearity, the variables pregnancy and number of children were mutually excluded from the fully adjusted models.

A strong inverse association was observed between breast cancer risk and number of children. Compared to nulliparous women, crude odds ratios were 0.90, 0.84 and 0.68 in women with one, two and three children respectively; p-value for trend < 0.01. However, as expected, there was a strong association between number of children and likelihood of breastfeeding. Nearly half of women with one child reported not having breastfed at all while only 10% of them breastfed for longer than 6 months. In contrast, amongst women with 3 or more children, only 15% did not breastfeed while as many as 38% reported breastfeeding for at least one year. The Kendall's tau-b was 0.42, p-value for independence < 0.001, indicating a relatively strong and statistically significant correlation between breastfeeding duration and number of children. Considering every possible pair among the participants, this non-parametric measure is calculated on the basis of the number of occasions where the ranking of both breastfeeding duration and number of children are in the expected direction (i.e. a higher rank in one variable is accompanied by a higher rank in the other) as compared to the number of discordant pairs. The strong univariable association observed with number of children attenuated once breastfeeding was controlled for in the multivariable logistic analysis while not much change was observed in the case of breastfeeding. This cannot be simply a result of collinearity as the confidence intervals also remained largely unaffected, more likely indicating that the observed association with number of children was confounded by the effect of breastfeeding.

In fact, this is also supported from the results of the stratified analysis where the estimate for the odds ratio for a one unit increase in the number of children was 0.92 (95%CI 0.80-1.05) among women who did not breastfeed and 0.97 (95%CI 0.82-1.15) among women who breastfed. The Mantel-Haenszel estimate controlling for breastfeeding is 0.94 (95% CI 0.85, 1.04) and test of homogeneity of ORs = 0.61, indicating no evidence of effect modification in the relationship of breast cancer and number of children by breastfeeding status. Further adjusting for other variables revealed no association with parity while interestingly wide confidence intervals were observed. On the other hand, the effect estimate of breastfeeding (and the confidence intervals for that matter) practically remained unchanged at 0.72 (95%CI 0.58, 0.90).

Table 3 presents odds ratios (and 95% CI) for breast cancer in terms of each participant characteristic before and after adjusting for the effect of all other in a multivariable model. On the basis of the results presented above, breastfeeding was included as a binary variable while parity was not considered further in the multivariable models. The strongest associations were observed with family history of breast cancer, age at menarche and breastfeeding. In fact, these associations persisted after adjusting for the effect of all other factors. For instance, the adjusted odds ratio for family history was 1.64 (95% CI 1.23-2.19) while for breastfeeding this figure was 0.74 (95% CI 0.59-0.92). Finally, a statistically significant trend with age at menarche and breast cancer risk was also observed. Women who started menstruating earlier than the age of 11 had an increased risk for breast cancer compared to women who started menstruating later, even after adjusting for the effect of all other risk factors. Adjusted odds ratio in women who started menstruating between the ages 12-14 was 0.82 (95%CI 0.62-1.10) and 0.64 (95%CI 0.45-0.82) in those who started after the age of 15 years; p-value for linear trend < 0.01.

**Table 3:** Odds ratios (95% CI) of breast cancer by participants characteristics before and after adjusting for the effect of all other in a multivariable model.

Risk factor		**Univariable**^**1**^		**Multivariable (N = 2020)**^**2, 6**^	
		
		OR (95%CI)	**p-value**^**3**^	OR (95% CI)	**p-value**^**3**^
Education	Primary	1.00			
	Secondary	1.17 (0.96,1.45)		1.18 (0.94,1.48)	
	Tertiary	1.09 (0.81,1.45)		1.12 (0.81,1.53)	
	University	1.35 (1.00,1.82)	0.07	1.40 (1.00,1,95)	0.08

Marital status	Married	1.00			
	Widowed/Divorced	1.08 (0.82,1.42)	0.57	1.06 (0.78,1.44)	0.84
	Single	1.55 (1.02,2.34)	0.04	1.28 (0.69,2.35)	0.43

BMI	<25	1.00			
	25-30	0.80 (0.64,0.98)		0.86 (0.69,1.07)	
	≥30	1.21 (0.96,1.53)	0.11*	1.22 (0.95,1.56)	0.14*

Family history of breast cancer	No	1.00			
	Yes	1.68 (1.29,2.19)	<0.001	1.64 (1.23,2.19)	0.001

Age at menarche (years)	≤11	1.00			
	12-14	0.80 (0.62,1.03)		0.82 (0.62,1.10)	
	≥15	0.61 (0.44,0.84)	<0.01	0.64 (0.45,0.92)	0.01

Age at 1st pregnancy (years)	<30	1.00			
	≥30	1.36 (1.04,1.77)		1.25 (0.93,1.68)	0.14
	Never^4^	1.51 (1.08,2.12)	<0.01	0.93 (0.57,1.54)	0.76

Breastfeeding^5^	No	1.00			
	Yes	0.63 (0.45,0.89)	<0.01	0.74 (0.59,0.92)	0.01

Oral contraceptive use	No	1.00			
	Yes	1.03 (0.86,1.26)	0.70	1.02 (0.83,1.27)	0.84

HRT use (months)	Never	1.00			
	<6	0.63 (0.42,0.95)		0.67 (0.42,1.05)	
	6-60	0.49 (0.37,0.65)		0.47 (0.35,0.64)	
	≥60	0.50 (0.34,0.72)	<0.001	0.53 (0.35,0.79)	<0.001

Exercise	No	1.00			
	Yes	0.86 (0.72,1.01)	0.07	0.94 (0.78, 1.13)	0.48

Smoking	Never	1.00			
	Past/Current	1.24 (1.01,1.53)	0.05	1.16 (0.91,1.47)	0.23

**Notes**: ^1^In all cases, models were adjusted for age. Both univariable and multivariable inferences remain largely unaffected when restricting the analyses to ages 45-64. ^2^Further adjusting for the effect of all other risk factors considered here. ^3^P-value for difference between levels of binary/nominal variables (e.g. family history) or p-value for linear trend across ordinal categorical variables (e.g. age at menarche or age at 1^st ^pregnancy). ^4^The variable 'pregnancy' was not included in the multivariable model as its effect is captured accordingly by category 'never' of the variable "age at 1^st ^pregnancy". ^5^Odds ratio of the combined effect of breastfeeding after adjusting for pregnancy in a logistic model with an interaction term between the two variables but no main effect for breastfeeding. ^6^The p-value of the Pearson χ^2 ^goodness-of-fit test was 0.18 in the case of the full model and 0.55 for the model where only statistically significant variables were included (i.e. family history, breastfeeding, age at menarche and HRT use), in both cases suggesting a reasonable fit of the model *Evidence of non-linearity based on Likelihood Ratio Test comparing the goodness of fit of model with ordered categories Vs linear term across categories.

None or only weak associations were observed between breast cancer risk and other risk factors investigated, for instance BMI, age at first pregnancy, smoking, exercise or use of oral contraceptives. Surprisingly, more controls (28.8%) than cases (15.5%) reported taking hormone replacement therapy (HRT). The inverse association persisted in the fully adjusted model, and in fact risk of breast cancer appeared to be inversely related to the duration of HRT use. Adjusting for number of children in the multivariable models, did not affect any of the estimates. Indicatively, the odds ratio for family history now becomes 1.62 (95%CI 1.22, 2.16), while the estimates were 0.83 (95% 0.62, 1.09) and 0.67 (95% 0.47, 0.94) in women who reached menarche between the ages of 12-14 and 15 or later respectively. Lastly, inferences were also unaffected when breastfeeding was expressed in terms of duration in the multivariable models (instead of the binary classification presented in the table).

## Discussion

The aetiology of breast cancer is still poorly understood and known breast cancer risk factors explain only a small proportion of cases. Epidemiological studies conducted in different populations have identified a spectrum of well established and probable risk factors for breast cancer [15]. These include age, socioeconomic status, reproductive events, breastfeeding, family history and lifestyle among others. However, most epidemiological breast cancer studies involve subjects living in North America and Western Europe, regions which represent only a fraction of the global population. Therefore there is a need to study breast cancer epidemiology in populations in less-well studied regions of the world, in order to gain a better understanding of breast cancer aetiology [15]. It is noted that this is the first epidemiological study into breast cancer risk factors in the Cypriot population.

The main aim of this study was to assess the strength of associations between some recognized risk factors and breast cancer among Cypriot women. We should note that while the factors investigated here (other than BMI) were all self-reported, there is no reason to believe that any recall should be substantially differential, not least due to the standardised interview procedure by which information was collected from both groups of participants. A total of 1109 Cypriot women diagnosed with breast cancer between years 1999-2005 were recruited. It should be noted that the incidence of breast cancer in Cyprus is now around 400 cases per year. The cases in this study represents as many as 50% of the total number of cases diagnosed between 1999-2005. As many as 90% of all breast cancer cases in Cyprus are registered, receive treatment and are followed by the Bank of Cyprus Oncology Centre in Nicosia. This centralized point of access, ensures that the study participants, are to a large extent representative of all cases on the island, in terms of their socio-demographic characteristics. It should be noted that the recruitment of cases was based on the list of scheduled appointments during the study period and not on a random selection of all diagnosed cases.

The 1177 controls were recruited amongst women who participated in the National breast cancer screening programme with the use of mammography, to a large extent the same population that would give rise to the cases. Since no matching was employed, unavoidable (small) differences in the age distribution between the cases and the controls (mainly due to the under-representation of younger and older age-groups amongst the mammography screening users) were dealt with by adjusting for age in multivariable analyses. The Cypriot National screening programme for breast cancer in all women aged 50-69 was officially introduced in 2006, after being pilot tested at a small-scale in 2003. Recent data from the Cyprus Ministry of Health suggest that more than half of the women invited respond to the call. Even so this does not imply that the sample of controls are representative of the general female population on the island, in terms of the background socio-economic characteristics and levels of potential risk factors for breast cancer in the general population.

In terms of the basic demographic characteristics of the two groups, no major differences were detected in the levels of education or marital status. However, well established risk factors for breast cancer identified in other populations, such as family history of breast cancer, age at menarche and breastfeeding exhibited the strongest associations with breast cancer risk among Cypriot women [4,5,10]. Unlike Britain and other European countries, there are no formal occupation-based socio-economic classification systems in Cyprus. In the absence of such socio-economic indicators or information on family income, educational status was used as a proxy. Controlling for the possible confounding effect of educational status in multivariable models did not affect inferences about major risk factors of breast cancer.

Healthy volunteers who participate in epidemiological studies tend to have a higher educational level, compared to the rest of the population [16]. In general, no major differences in the level of education between cases and controls were observed in this study. Nevertheless, it is possible that women who attend the screening programme are likely to be more educated, thus masking any such differences. Furthermore, a study of risk factors of breast cancer in Iran has shown that marital status may have an impact on the incidence of breast cancer in Iranian women. It was observed that women who never married were at higher risk for breast cancer [17]. However, marital status by itself may not be a determining factor for modifying breast cancer risk. Since there is a strong interaction between marital status and parity, the increased breast cancer risk associated with single women may possibly be due to nulliparity.

Many studies have examined the association between body mass index (BMI) and breast cancer incidence. It was observed that in postmenopausal women, obesity (BMI > 30 kg/m^2^) was associated with about 50% increase in breast cancer risk when compared with lean women (BMI 20 kg/m^2^). This association was not observed in premenopausal women. In contrast, in some studies it was observed that during the premenopausal years, breast cancer risk was slightly lower in obese women [18]. In our study, no information on menopause status at the time of diagnosis was available. By comparing BMI at the time of interview between cases and controls, no statistically significant differences were observed between the two groups. The relationship between BMI and breast cancer risk is complex and is better assessed in prospective studies where further anthropometric data are accurately collected at baseline and at regular intervals afterwards. In this context, no conclusive results regarding the association between BMI and breast cancer incidence in the Cypriot population can be drawn on the basis of this study.

Family history of breast cancer is one of the most well established, widely accepted risk factors for this disease. Having one first-degree relative with breast cancer approximately doubles a woman's risk for developing breast cancer. The risk is elevated significantly by increasing the number of affected relatives [19,20]. Our findings are in accordance with other published studies and suggest that a positive family history of breast cancer is one of the most significant risk factors for this disease in Cyprus. In fact, if women with positive family history of cancer are more likely to attend mammography clinics for screening, the observed association may actually be an underestimation.

Breast cancer risk is associated with several reproductive factors. It is well established that breast cancer risk increases with early age at menarche [21-23]. This association is consistent with the hypothesis that breast cancer risk is related to the extent of breast mitotic activity. This activity is driven by estrogen and progesterone exposure during the luteal phase of the menstrual cycle [24], which determines the probability of tumorigenic somatic events [25]. Therefore, an early age at menarche increases the period during which the breast is mitotically active and subsequently increases breast cancer risk. Similarly to previous investigators, we observed that an early age at menarche is associated with an elevated risk of breast cancer in our population.

The effect of breastfeeding on breast cancer risk has been controversial indicating either no association, or a weak protective effect against breast cancer [26]. Studies in countries with a long duration of breastfeeding have reported substantial protective effects, whereas a number of studies in Western populations failed to detect an association, possibly due to the low prevalence of prolonged breastfeeding [26]. A meta-analysis of breastfeeding and breast cancer risk, by the Collaborative Group on Hormonal Factors in Breast Cancer, showed that increasing duration of breastfeeding confers a protective effect on breast cancer risk, over and above that already known to be afforded by parity itself [27]. Breastfeeding is hypothesized to reduce the risk of breast cancer primarily through two mechanisms, differentiation of breast tissue [28] and reduction of the lifetime number of ovulatory cycles [29]. The results of our study suggest an inverse association between breastfeeding and breast cancer risk, even though a dose-response relationship with self-reported duration of breastfeeding was not observed, possibly a result of misclassification. This finding is consistent with the results of the large collaborative study showing breastfeeding to be protective for breast cancer [27]. Breastfeeding is one of the few potentially modifiable factors that can reduce breast cancer risk. Further investigations are warranted in order to understand the underlying mechanisms of the protective effect of breastfeeding and how protection might be conferred. Once these are determined, interventions, which would mimic breastfeeding, could be developed for the benefit of women who have never breastfed [27].

Surprisingly, we observed an inverse association between hormone replacement therapy (HRT) and breast cancer risk. Results from randomized controlled trials and from observational studies have shown that use of HRT increases breast cancer risk. Furthermore, the effect of HRT on breast cancer risk depends also on the different combinations of HRT as well as on the duration of usage [30-34]. Overall, studies have concluded that oestrogen-progestagen combinations increase the risk of breast cancer, and that the risk was elevated, in women who were treated for at least 5 years [32,34]. The above results should be interpreted with caution due to the difficulties experienced by Cypriot women in recalling information regarding type and exact duration of HRT use. However, there is no reason to believe that such recall was differential among cases and controls. Unlike other studies [34,35], the vast majority of women in our sample reported never having used HRT while only a small percentage of women in our sample reported use for periods longer than 5 years (e.g. 7.8% among healthy controls). Furthermore, while in prospective studies one would expect to find an association between HRT use and breast cancer risk, in our retrospective study it is not surprising to observe an inverse association. It is very likely though that cases, once diagnosed with breast cancer, were not prescribed with HRT which would explain the reverse association seen in our study.

Only a weak association was observed between lifestyle risk factors such as exercise and smoking. It should be noted though that these parameters refer to current status only, as no information for past habits, duration or intensity of smoking were available. As a result, any association is hard to interpret due to problems with directionality of effect e.g. associations with exercise may be more likely to reflect that women diagnosed with breast cancer are less likely to currently exercise, rather than a causal relationship between lack of exercise and breast cancer.

## Conclusions

The strongest associations with breast cancer risk in the Cypriot population were observed with family history of breast cancer, early menarche and breastfeeding. It is noteworthy that the protective effect of breastfeeding appeared stronger than the effect of pregnancy alone. Overall, the findings of our study corroborate with the results of previous investigations on descriptive epidemiology of risk factors for breast cancer. Nevertheless, this study presents the first report for breast cancer risk factors in Cypriot women and provides the first scientific evidence using National data, for executing more targeted campaigns of prevention and early diagnosis in the Cypriot population.

## Competing interests

The authors declare that they have no competing interests.

## Authors' contributions

AH participated in the conception and design of the study, in obtaining funding, as well as the collection, assembly, analysis and interpretation of data. ML participated in the conception and design of the study, the collection, assembly, analysis and interpretation of data as well as prepared the first draft of the manuscript with KK. NM offered guidance on statistical matters, performed statistical analysis and contributed towards the interpretation and presentation of the data in the manuscript. TM and RP participated in collection and assembly of data. EK, MD, PP, SM and YM provided patients and participated in data collection and interpretation. KK participated in the conception and design of the study, in obtaining funding, collection and assembly of data, analysis and interpretation of data and drafted the manuscript. All authors read and approved the final manuscript.

## Pre-publication history

The pre-publication history for this paper can be accessed here:

http://www.biomedcentral.com/1471-2407/10/447/prepub
